# Dodecamer d-AGATCTAGATCT and a Homologous Hairpin form Triplex in the Presence of Peptide REWER

**DOI:** 10.1371/journal.pone.0065010

**Published:** 2013-05-21

**Authors:** Amrita Das, Tapas Saha, Faizan Ahmad, Kunal B. Roy, Vikas Rishi

**Affiliations:** 1 Department of Chemistry, University of Kolkata, Kolkata, West Bengal, India; 2 Georgetown University, Washington D.C., United States of America; 3 Centre for Interdisciplinary Research in Basic Sciences, Jamia Milia Islamia, New Delhi, India; 4 Center for Biotechnology, Jawaharlal Nehru University, New Delhi, India; 5 National Agri-Food Biotechnology Institute (NABI), Mohali, Punjab, India; Institut Pasteur, France

## Abstract

We have designed a dodecamer d-AGATCTAGATCT (RY12) with alternate oligopurines and oligopyrimidines tracts and its homologous 28 bp hairpin oligomer (RY28) that forms a triple helix only in the presence of a pentapeptide REWER. An intermolecular triplex is formed by the single strand invasion of the RY28 duplex by RY12 in the presence of REWER. 5′- oligopurine end of RY12 binds to oligopurine sequence of RY28 in a parallel orientation and its oligopyrimidine stretch then changes strand and adopts an antiparallel orientation with the other strand of the duplex. Evidence for the formation of the triplex come from our studies of the UV melting curves, UV mixing curves, gel retardation assay, and chemical sequencing of 1∶1 mixture of dodecamer and hairpin oligonucleotides in the presence and absence of the peptide REWER. RY12 exists as a duplex that melts at 35°C. The hairpin (RY28) melts at 68°C. 1∶1 mixture of RY12 and RY28 in the absence of REWER gives a biphasic transition curve with thermodynamic properties corresponding to those of the melting of the duplex of RY12 and the hairpin RY28. However, the melting curve of this mixture is triphasic in the presence of the REWER; the thermodynamic parameters associated with the first phase (melting of the duplex of RY12), second phase (melting of the triplex) and the third phase (melting of the hairpin) show dependence on the molar ratio of peptide to oligonucleotides. Under appropriate conditions, gel retardation assay showed a shifted band that corresponds to a possible triplex. Chemical sequencing of KMnO_4_ and DEPC treated mixture of RY12, RY28 and REWER revealed the footprint of triplex.

## Introduction

The considerable interest in DNA triple helix arose due to its role in human diseases [1], [2] and its potential use in targeted gene therapy [3]–[5]. Triplex-forming oligonucleotides (TFO) can be designed that recognizes duplex DNA target in a sequence-specific manner. Studies have shown that intramolecular triplexes in which a single strand of DNA folds back into a triple-helical structure can form *in vivo* [6]. Purine stretches in the double helix are essential for triplex helix because pyrimidine do not have enough donor and acceptor groups to form both Watson-Crick and Hoogsteen hydrogen bonds simultaneously. Several groups used designed deoxyribonucleotides to demonstrate that intermolecular triplexes where the third strand occupies the major groove of the duplex could form at sites that are purine-rich in one strand. In these triplexes the third strand can be either parallel or antiparallel. In Pu•PuPy triplex type purine-rich third strand is hydrogen bonded to underlying purine strand of duplex in antiparallel orientation whereas pyrimidine-rich third strand in Py•PuPy triplex forms parallel triplex with purine strand of duplex. In third type of triple-helical structure the third strand pair first with purines on one strand and then switches strand to pair with purines on the other strand [6]. Recombination triplexes that form *in vivo* in the presence of proteins like RecA in bacteria [7] and RAD51 in eukaryotes [8] are different from the conventional triplexes observed experimentally. Contrary to the known antiparallel triplexes the third strand here is accommodated in the major groove of DNA duplex in parallel orientation of like strands with deoxyriboses in north conformation [9], [10]. Several models of major groove associated parallel triplex structure have been proposed [11], [12].

In a previous study an antiparallel triplex formation between synthetic deoxyoligonucleotide hairpin RY28 molecule with a homologous dodecamer YR12 was reported [13]. It was shown that RY28 does not form triplex structure with RY12, that has the same sequence but opposite polarity to that of YR12 dodecamer [13]. Authors showed that a spontaneous triplex formation is permitted only when identical strands are oriented antiparallel but not permitted in parallel orientation as in R-form DNA. Due to chosen sequences the triplex between RY28 and RY12 would be parallel in Pu•PuPy and antiparallel in Py•PuPy. Such parallel triplex is formed only under forced strand orientation or with the assistance of a protein like RecA [14]. Our synthetic oligomers (RY12 and RY28) contain the same alternate repeats of three purines and three pyrimidines so that all the strands are homologous. Any triplex formed between two oligonucleotides must involve pairing with both the strands of Watson-Crick (W-C) duplex. We now report here the evidence for the formation of a triplex between RY12 and RY28 mediated by a designed pentapeptide, Arg-Glu-Trp-Glu-Arg (REWER).

## Materials and Methods

### Oligonucleotides and peptide synthesis

Synthesis and purification of RY12 (5′-AGATCTAGATCT-3′) and RY28 (5′- AGATCTAGATCTCTTCAGATCTAGATCT-3′) deoxyoligonucleotides were carried by solid phase β-cyanoethylphosphoramidite chemistry [15]. Deprotection and purification of the dodecamer oligonucleotides were done by standard procedures [16]. We designed peptide REWER such that the tryptophan is placed in the middle of the pentapeptide and there are two glutamic acids and two arginines flanking it making this peptide a charge-neutral molecule. The pentapeptide, REWER was synthesized by solid phase peptide synthesis using F-moc chemistry on a semi-manual peptide synthesizer (Novasyn, France). The crude peptide was purified on reverse phase column (GE Healthcare, USA). The peptide synthesis reagents were from Novabiochem (USA). All solvents and reagents were of analytical grade from Sigma Chemical Co. (USA). Stock solutions of oligonucleotides RY12 and RY28 and peptide, REWER were prepared in Buffer A (10 mM Na-cacodylate buffer (pH 5.8) with 100 mM NaCl and 10 mM MgCl_2_). Concentrations of the stock solutions of REWER, RY28 and RY12 were determined using absorption coefficient (M^−1^ cm^−1^) values of 5,700, 9,000, and 10,000, respectively.

### Optical studies

For spectroscopy measurements samples with appropriate concentration of oligonucleotides and peptide (4.5×10^−5^–5.5×10^−5^ M) were prepared in degassed buffer. Thermal denaturation measurements were carried out in JASCO UV-Vis 560 spectrophotometer with a heating rate of 1°C per minute using a Peltier accessory (Model ETC-505). The change in absorbance of each sample was measured at 260 nm, and approximately 350 data points were collected for each denaturation curve. Prior to melting study of oligomer mixtures, solutions were heated to 90°C for 2 minutes and annealed by slowly decreasing the temperature to 0°C. We found that these annealing conditions facilitated formation of the interacting complex and resulted in a well-defined melting profile. The reversibility of the transition was checked by matching the absorbance at 260 nm and 25°C before and after heating.

For UV mixing experiments equimolar (30 µM each) mixture of RY12 and RY28 were prepared in the degassed Buffer A. Each solution contained the peptide REWER and nucleotides at a definite [P]/[N] ratio where [P] and [N] represent the initial molar concentration of the peptide and total DNA strand concentration, respectively. 50 µl aliquot of one oligonucleotide solution was added stepwise to 400 µl of stock solution of the other oligonucleotide contained in a stopper cuvette [17]. After each addition the cuvette was inverted repeatedly to ensure complete mixing followed by heating at 90°C for one minute and incubation at room temperature for 20 minutes. Absorbance was then recorded at 260 nm. The results given are average of three absorbance measurements at a specific molar ratio.

### Gel retardation assay

Gel retardation assay was carried out by labeling the 5′end of either RY12 or RY28 oligomer using T_4_ polynucleotide kinase (New England Biolabs (NEB), USA) and [γ^32^P]-ATP (GE Healthcare, USA) using the procedure described elsewhere [18]. The radiolabeled RY28 or RY12 oligomer was mixed with the other cold oligomer in Buffer A. Solutions were made as described for UV mixing experiments. Each sample contained peptide at a ratio of [P]/[N] = 3.8. All solutions in Buffer A were heated to 90°C for two minutes, followed by slow annealing at room temperature. 5 µl of calf thymus DNA (CTD) (1 mg/ml), 100 µl of 0.5 M NH_4_(OAc) and four volumes of ethanol were added to each sample to precipitate the complex overnight at −70°C. The precipitates were washed twice with 70% ethanol followed by drying. The samples were then dissolved in loading dye and immediately resolved on 15% native polyacrylamide gel (PAGE) that contained excess amount of free REWER in the gel itself. PAGE was run at 150 V in 0.25× TBE buffer for 3 hours in a cold room. The same experiment was also performed using labeled RY12 and unlabeled RY28. Since the precipitation of 12 nt DNA is difficult, radiolabeled RY12 oligomer was precipitated in the presence 20 µl of CTD (1 mg/ml) and 200 µl of 0.5 M NH_4_(OAc) and four volumes of ethanol.

### Sequencing of Triplex structure

Chemical modifications using potassium permanganate (KMnO_4_) (Sigma Chemical Co., USA) and diethyl pyrocarbonate (DEPC) were carried out at [P]/[N] ratio of 3.8. KMnO_4_ sequencing reactions were carried out on free RY28 oligomer and RY28-RY12 complex in the presence and absence of peptide REWER as per published procedure with slight modifications [19]. Approximately 300 ng of RY28 oligomer was radiolabeled with [γ^32^P] ATP at the 5′ end. All samples were dissolved in the reaction Buffer A, heated to 90°C and then slowly annealed at room temperature. 5 µl of 10 mM KMnO_4_ diluted freshly from the 100 mM stock at 4°C, was then added and the reaction was continued for 10 minutes at 4°C. After chemical modifications, each oligonucleotide sample was precipitated by ethanol in the presence of high salt and then subjected to pyrrolidine cleavage [20], followed by loading on a 20% denaturing sequencing PAGE containing 8% urea. Bands were resolved by gel electrophoresis at 1200 V for 3 hours. For DEPC modification, 5′ end radiolabeled RY28 oligonucleotide solution in the reaction Buffer A was cooled to 4°C and then 5 µl of DEPC (Sigma-Aldrich, USA) was added and incubated at 4°C for 30 minutes. The subsequent precipitation, pyrrolidine cleavage and electrophoresis on 20% denaturing PAGE with 8% urea was done as described for KMnO_4_ reaction.

## Results

### Analysis of UV thermal transitions

**Figure 1:**
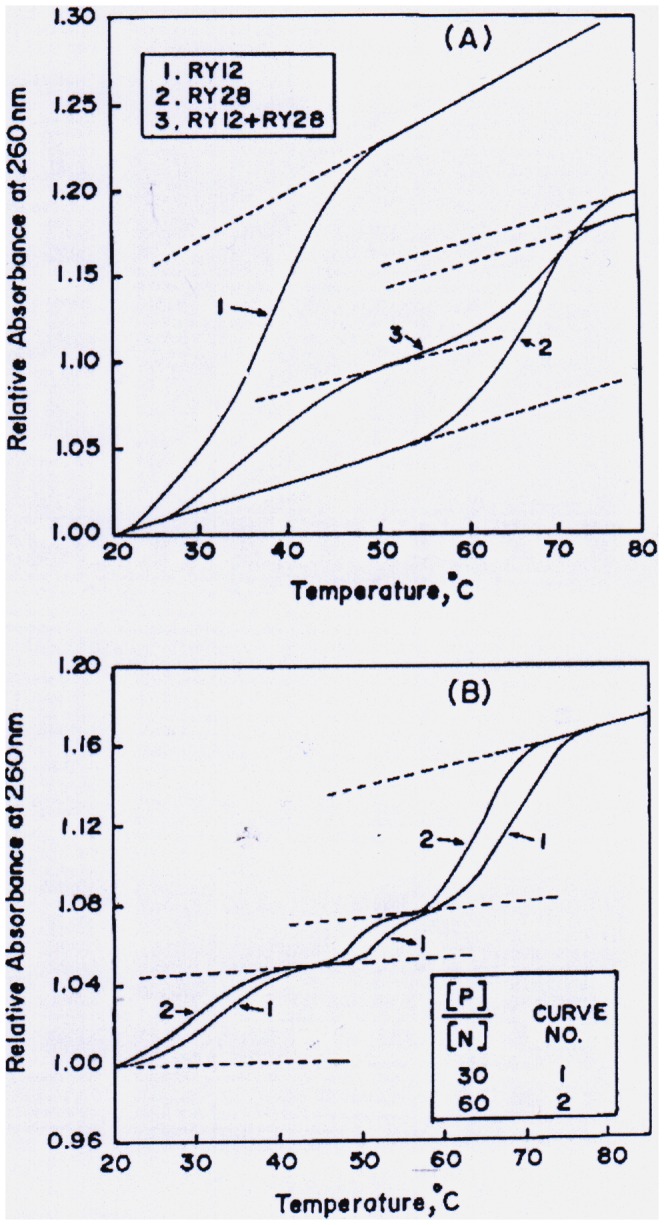
Melting profiles of oligonucleotides RY12 and RY28 and their mixtures in the presence and absence of pentapeptide REWER. **A**) Heat-induced denaturation of the RY12 duplex (curve 1), RY28 hairpin (curve 2) and equimolar mixture of RY12 and RY28. **B**) Melting curves of equimolar mixtures of RY12 and RY28 in the presence of the peptide REWER at the [P]/[N] ratio of 30 (curve 1) and 60 (curve 2). To maintain clarity melting curves at other [P]/[N] ratios are not shown.

Figure 1A (curve 1) shows the melting of the RY12 duplex. This process is reversible. Assuming a two-state mechanism, ΔG_d_(T), the Gibbs energy change at any temperature *T* K, associated with the melting of the duplex (double strand (d)↔2 s (single strand)) is determined with the help of following relation,
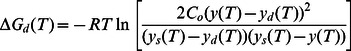
(1)where *R* is the gas constant; *C*_o_ is the total strand concentration; *y*(T) is the absorption at T K; and *y*_d_(T) and *y*_s_(T) are the optical properties of the duplex (pretransition region) and single strand (posttransition region) at T K, respectively. Values of Δ*G*_d_(T) in the range −1.3<ΔG_d_(T), kcal mol^−1^<1.3 were plotted as a function of temperature. This plot was used to determine T_m_^d^ (midpoint of melting transition of the duplex) and ΔH_m_^d^ (enthalpy change on melting of the duplex at T_m_^d^) using the procedure described elsewhere [21]. These values are given in Table 1.

**Table 1:** Thermodynamic parameters associated with the melting transitions of the duplex, hairpin and triplex^1^^,^ 2.

Compound	*T*_m_^d^	Δ*H*_m_^d^	*T*_m_^t^	Δ*H*_m_^t^	*T*_m_^h^	Δ*H*_m_^h^
RY12 (-Pep)^3^	35.0±0.1	45.0±2.0	_	_	_	_
RY28 (-Pep)	_	_	_	_	68.0±0.2	75.0±5.0
RY12+RY28 (-Pep)	35.1±0.1	45.0±3.0	_	_	68.8±0.1	80.3±2.0
[P]/[N] = 15	35.3±0.2	45.6±1.0	54.5±0.1	142.0±9.0	69.5±0.1	96.8±2.0
[P]/[N] = 30	35.7±0.1	52.5±2.0	53.7±0.1	148.0±8.0	69.9±0.1	98.0±3.0
[P]/[N] = 45	32.1±0.1	43.7±1.0	52.5±0.1	98.9±3.0	65.5±0.1	89.0±1.0
[P]/[N] = 60	29.9±0.4	42.0±1.0	50.3±0.2	102.0±4.0	65.1±0.1	83.0±1.0

1 Superscripts d, h and t represent duplex, hairpin and triplex, respectively.

2 A ‘±’gives the mean error of the triplicate measurements. The maximum standard errors of the least-squares analysis of the stability curve are ±0.5°C and ±5 kcal mol^−1^ for T_m_ and ΔH_m_, respectively.

3 (-Pep) measured in the absence of the pentapeptide.

Figure 1A (curve 2) shows the melting transition curve of hairpin RY28. This process (native hairpin (h_n_) state↔unfolded hairpin (h_u_)) state is reversible. Assuming a two-state mechanism, ΔG_h_(T), the Gibbs free energy change at any temperature T K, associated with the melting of the hairpin is determined with the help of the relation,
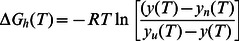
(2)where *y*(T) is the absorption at T K, and *y*_n_(T) is the absorption of the native hairpin (pretransition) and *y*_u_(T) is the absorption of unfolded (posttransition region) forms of the hairpin. Values of ΔG_h_(T) in the range −1.3<ΔG_d_(T), kcal mol^−1^<1.3 were determined. These values of ΔG_h_(*T*) were plotted as a function of temperature and were used to determine T_m_^h^ and ΔH_m_^h^ as described above. All such values are given in Table1.

Figure 1A shows the melting transition curve of 1∶1 mixture of RY12 and RY28 (curve 3). This melting curve is biphasic and reversible. RY12 and RY28 do not interact and melt independent of each other. Assuming that the first phase (i.e., the process in the lower temperature range) represents the melting of the duplex (RY12) and the second phase (i.e., the process in the higher temperature range) is the unfolding of the hairpin (RY28), they were analyzed for T_m_^d^, ΔH_m_^d^, T_m_^h^, and ΔH_m_^h^ using equation 1 and equation 2 and the methods described above. It should be noted that the posttransition region of the first phase is the pretransition region of the second phase. Thermodynamic parameters are given in Table 1.

We also measured heat-induced transitions of the 1∶1 mixture of RY12 and RY28 in the presence of peptide REWER at different mole ratios of [P] and [N], where square brackets represent the molar concentrations of the peptide (P) and the nucleotide (N), respectively. All transitions were reversible and triphasic. Figure 1B shows representative transition curves of mixtures. It is assumed that the first and the third phases represent melting of the duplex and hairpin, respectively, and they were analyzed for (T_m_^d^ and ΔH_m_^d^) and (T_m_^h^ and ΔH_m_^h^) values as described above. These values at different [P]/[N] ratios are shown in Table 1. The intermediate phase of the transition shown in Figure 1B were analyzed for ΔG_t_(T), the Gibbs free energy change associated with the melting of the triplex, h_n_−s↔h_n_+s, using the following equation,
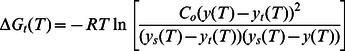
(3)where *C*_o_ is the total strand concentration, y(T) is the observed optical property for the intermediate phase at T K, y_s_ is the optical property of the single strand when it is dissociated from the triplex (i.e., in the post transition region of the melting of the triplex) and shows a small temperature-dependence. y_t_ is the optical property of the triplex (i.e., the complex of hairpin in the native state and the single strand existing in the posttransition region of the first phase. y_t_ also shows a small temperature-dependence. Stability curve (ΔG_t_(T) versus T) were drawn at each [P]/[N] ratio as described above and analyzed for T_m_^t^ (midpoint of melting of triplex) and ΔH_m_^t^ (enthalpy change at T_m_^t^). Values of T_m_^t^ and ΔH_m_^t^ at different [P]/[N] ratios are given in Table 1.

### UV mixing curve determines the stoichiometry of interaction between RY12 and RY28

**Figure 2:**
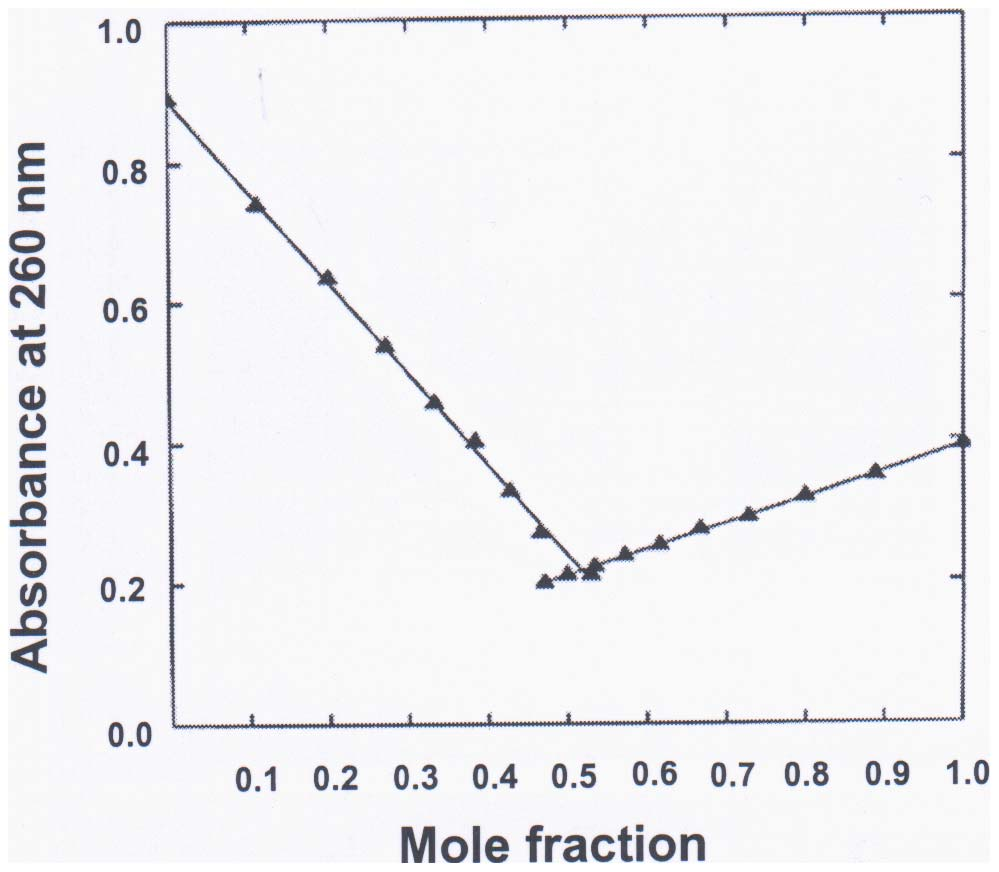
UV mixing curves show the stoichiometry of interaction between RY28 and RY12 in the presence of REWER. Both the oligonucleotide solutions contained REWER at [P]/[N] ratio of 30 prior to mixing. The abscissa is in mole fraction of RY28. There is a clear inflection point at 0.5 mole fraction indicating that single strand of RY12 interact with RY28 hairpin.

If a single strand of RY12 combines with the hairpin RY28 to form a triplex structure, the stoichiometry of this interaction can be determined by UV mixing curve as shown in Figure 2. First order regression of the data points obtained in Job titration shows a clear inflection at 0.5 mole fraction in terms of strand concentration suggesting that complex formation occurs between one RY28 hairpin molecule and a single strand of RY12 in presence of REWER forming a three stranded structure.

### Gel retardation assay demonstrates the formation of triplex

**Figure 3:**
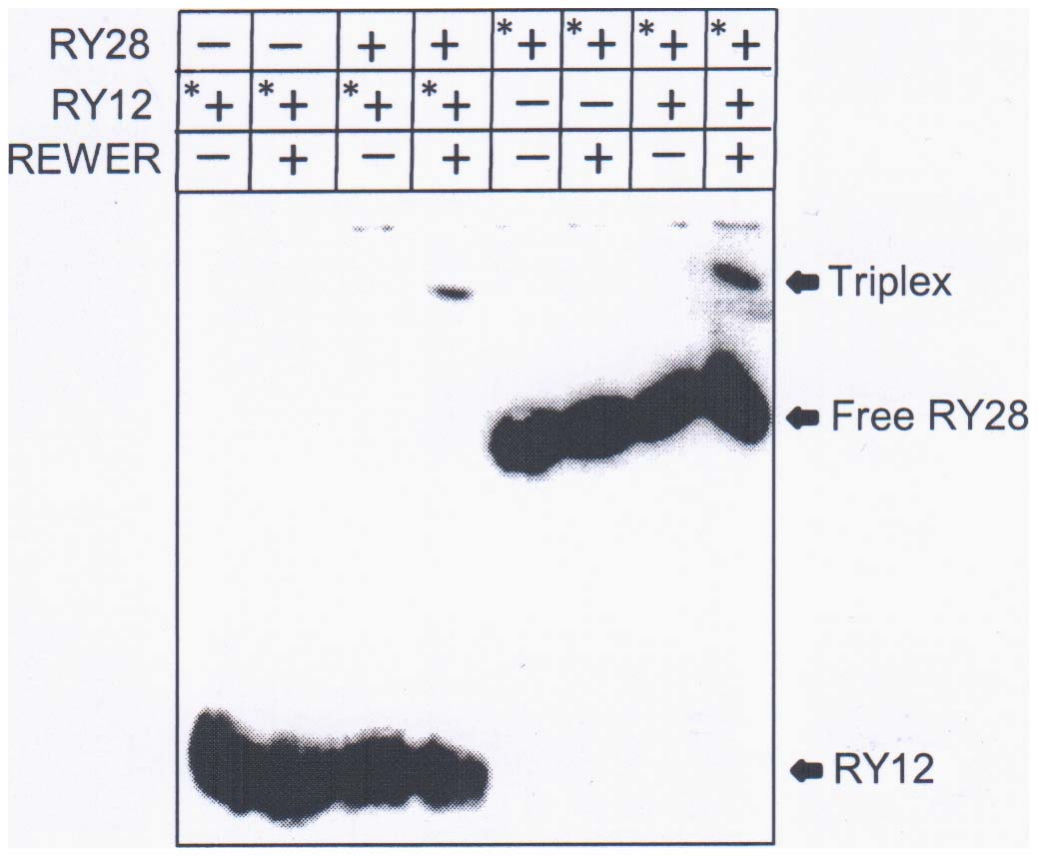
EMSA shows the formation of triplex between RY12 and RY28. Triple helix formation between dodecamer RY12 and RY28 hairpin in the presence of the peptide REWER detected on 15% native polyacrylamide gel electrophoresis. First four lanes show the results of experiments using radiolabeled RY12, last four lanes used radiolabeled RY28. * represents 5′ radiolabeled oligonucleotide.

The autoradiogram of the gel-electrophoresis of the free RY12, RY28 and their 1∶1 mixture in the presence and absence of the peptide REWER, are shown in Figure 3. Either of the oligomer was radiolabeled and all samples underwent same treatment before electrophoresis. A retarded band, indicative of the triplex is seen in the mixture of RY28 and RY12 only in the presence of the peptide. The same band is seen irrespective of whether RY12 or RY28 is labeled.

### Fine mapping of triplex by chemical probing

**Figure 4:**
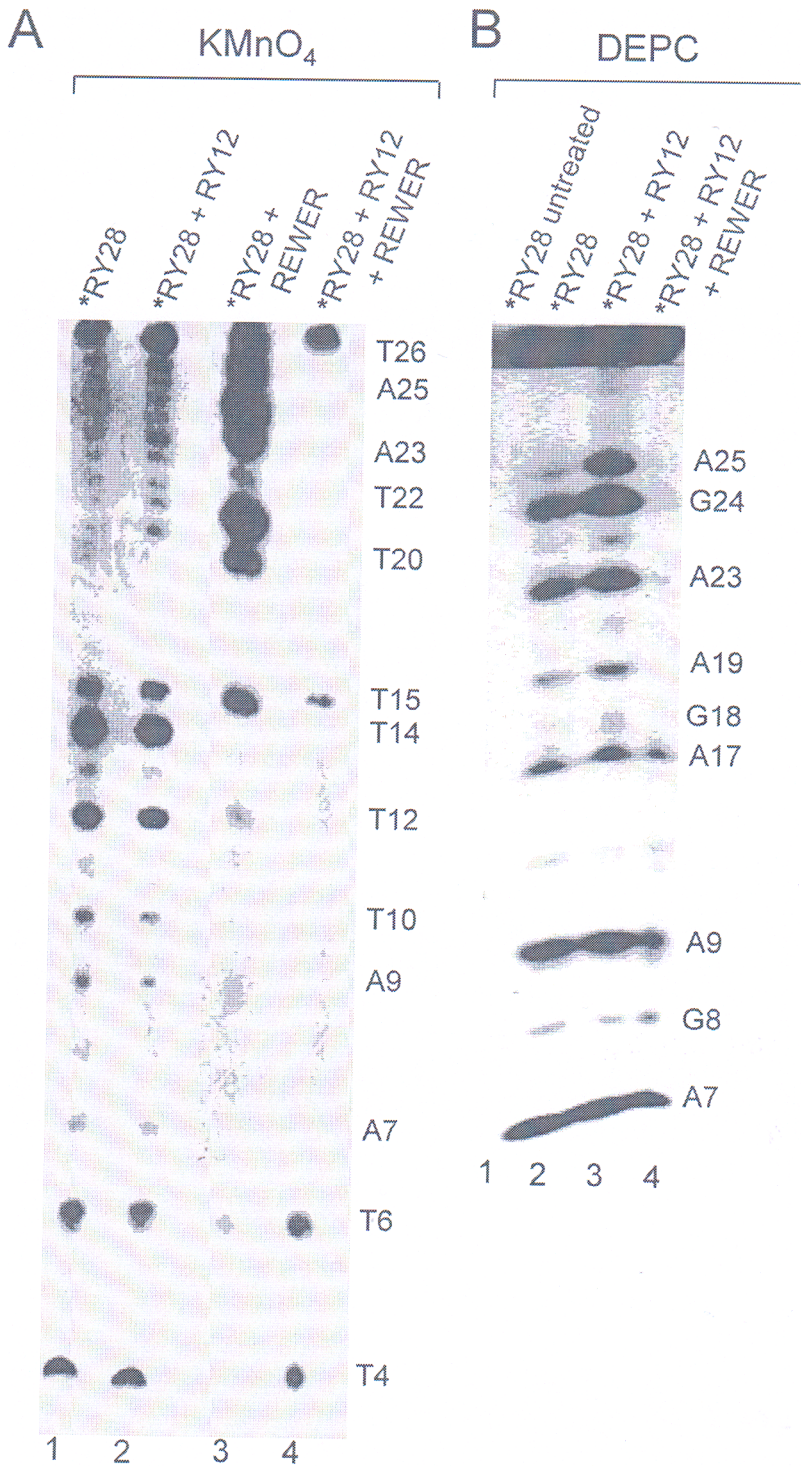
KMnO_4_ and DEPC sequencing results show protection of DNA bases due to triplex formation in the presence of REWER. Prior to electrophoresis all chemical modifications were done at 4°C. **A**) KMnO_4_ panel shows the reactivity of the free RY28 and RY28+RY12 (1∶1) mixture with radiolabeled RY28 in the presence and absence of the pentapeptide, REWER. [P]/[N] ratio in all experiments was 3.8. Lane1, RY28 alone; lane 2, RY28+RY12 mixture; Lane 3, RY28+REWER; lane 4, RY28+RY12+REWER. Bands corresponding to thymines and adenines are indicated. **B**) DEPC reactivity of the triplex. Lane 1 represents probe only (without treatment) and was used as control for both KMnO_4_ and DEPC experiments. Lane 2, RY28; lane 3, mixture of radiolabeled RY12+RY28; lane 4, RY28+RY12+REWER. The bands corresponding to adenines and guanines are marked. * represents 5′ radiolabeled oligonucleotide.

KMnO_4_ modifies exposed thymines at the C5–C6 double bond in single stranded or unwound region of nucleic acids [22], although purine modification has also been reported [23]. Results of KMnO_4_ modification on RY28 hairpin are shown in Figure 4A (lane 1). Surprisingly all the thymines of RY28 are sensitive to KMnO_4_ with T4, T6, T12, T14, and T15 being hypersensitive. The pattern is asymmetric and unusual and shows that the thymines of the 5′-half are hypersensitive while those of 3′-half are not. This pattern remains unchanged even after addition of RY12. Figure 4A, lane 2 shows the results of KMnO_4_ modification on a sample of 1∶1 mixture of labeled RY28 and unlabeled RY12. The pattern for RY28 alone is drastically altered when REWER is present in the annealed sample; the 3′-half of the hairpin becomes hypersensitive while 5′-half is protected from KMnO_4_ modification when the peptide is present in the sample. (Figure 4A, compare lane 3 with lanes 1 and 2). In the presence of REWER, 1∶1 mixture of RY28 and RY12 gave a footprint of triplex (lane 4). Thymines are now resistant to KMnO_4_ in the triplex, and protection is more pronounced at T26, T22, T20, T12 and T10. The overall reactivity pattern is consistent with A•A­T and T•A­T base triplets). Even thymines in the loop (T12 and T15) show little sensitivity. As in hairpin T4 is reactive in the triplex also possibly due to fraying ends.

DEPC is used as a DNA structural probe for detection of bases at B-Z junction [24] and at cruciform loop [25]. DEPC carboxylates purines at N7 [24]. Adenines in WC duplex show protection from DEPC modification when N7 is engaged in Hoogsteen pairing in normal antiparallel triplex [25]. The reactivity of RY28 towards DEPC is shown in Figure 4B (lane 2). All the adenines and guanines are not equally reactive. Bands of A25, G24, A23, A19, A17, A9, G8 and A7 are shown in lane 2. A similar band pattern is observed when DEPC reaction was carried out on a mixture of radiolabeled RY12 and RY28 in absence of peptide. Band corresponding to A25 is prominent (Figure 4B, lane3). Lane 4 shows that in the presence of REWER, however, 1∶1 mixture of RY12 and RY28 forms a triplex, shown by the band pattern after DEPC treatment. Intense bands of A25, G24, A23 and A19 disappear completely, while bands of A17, A9 and G8 persist, *albeit* with diminished intensities. This observation is consistent with the results of KMnO_4_ modification described above.

## Discussion

A prerequisite to an *in vivo* parallel triple helix between a single strand of DNA and the homologous duplex DNA is the localized unwinding of DNA double helix. Previous studies have used a pentapeptide (KGWGK) where tryptophan intercalates between bases and induces unwinding of DNA [26]. REWER with a centrally placed tryptophan is suggested to do the same first by unfolding the DNA and then enabling the invasion of third strand resulting in the formation of triple helix.

The first indication of triplex formation comes from the analysis of UV thermal transition of 1∶1 mixture of RY12 and RY28, in presence and absence of the pentapeptide REWER. The free oligomer RY12 in 100 mM NaCl exists as a duplex which melts cooperatively with T_m_ and ΔH_m_ values of 40°C and 66 kcal mol^−1^, respectively [26]. Under the present solution conditions the T_m_ and ΔH_m_ for RY12 melts are 35±0.1°C and 45±2 kcal mol^−1^, respectively. These lower values are possibly due to different buffer conditions used in the present study. The RY28 hairpin denaturation is also a cooperative process (Figure 1A). It is seen in Figure 1A that relative absorbance (A_rel_) increases linearly up to 55°C followed by a sigmoidal transition and then a linear increase above 78°C. We assume that the linear portions represent the pre-and posttransition region of a two-state, helix↔coil transition. Analysis of the transition curve gave T_m_ and ΔH_m_ values of 68±0.2°C and 75±5 kcal mol^−1^, respectively, for the RY28 hairpin. On heating 1∶1 mixture of RY28 and RY12, we observed a biphasic transition, where each curve represents the melting of individual oligonucleotide. Analysis of the biphasic curve gave values of T_m_ and ΔH_m_ that are close to those of RY12 duplex and RY28 hairpin individually (Table 1), suggesting that RY12 and RY28 do not interact under these conditions. These results are in agreement with those reported earlier [13]. Upon addition of the pentapeptide REWER to the 1∶1 mixture of RY12 and RY28, however, a triphasic transition was observed (Figure 1B). This additional phase transition appears in an intermediate temperature range, when the RY12 duplex is completely melted but the RY28 is intact. This raises the possibility of a single strand of RY12 interacting with the hairpin molecule to form a three-stranded structure with T_m_ = 54.5°C, which is incidentally higher than the T_m_ = 35°C of normal antiparallel triplex formed between RY28 and YR12 [13]. Thermodynamic analysis of triphasic curve in the presence of different peptide concentrations gave values of T_m_ and ΔH_m_, and these are included in Table 1. T_m_ and ΔH_m_ values associated with denaturation of the duplex and the hairpin increased slightly after addition of the peptide up to a [P]/[N] ratio of 30. A further addition of the peptide causes significant decrease in T_m_ and ΔH_m_ (Table 1). One possible explanation is that the addition of a positively charged peptide at low concentration initially neutralizes phosphate charges and stabilizes the duplex structure. The stabilizing effect is offset at higher concentration of REWER, when the peptide binds in the major groove of the duplex or hairpin stem and destabilizes both the RY12 and RY28 by intercalating tryptophan side chain [26]. The thermodynamic characteristics of the purported triplex are given in Table 1. It is interesting to note that before destabilizing conditions are reached (i.e., when triplex has not fully formed) ΔH_m_^t^ is the sum of ΔH_m_^d^ and ΔH_m_^h^. However, the values of ΔH_m_^t^ at the two highest peptide concentrations, when peptide binding and intercalation is complete, are less than the sum of ΔH_m_ values of RY12 and RY28 suggesting an enthalpy driven complex formation between the two components.

The stoichiometry of the interacting complex was determined by UV mixing curve in the presence of REWER. The inflection point at 0.5 mole fraction establishes the 1∶1 stoichiometry of RY12 and RY28 oligomers in the complex indicating a three-stranded structure (Figure 2). Detection of the triplex on the native PAGE under standard conditions proved difficult due to small size and low overall stability of the triplex. In order to encourage the triplex formation, samples were run in cold room with high concentration of the pentapeptide in the polyacrylamide gel itself. Under these conditions, 1∶1 mixture of RY12 and RY28 showed a shifted band that may corresponds to triplex (Figure 3). The same shifted band is observed irrespective of whether RY28 or RY12 is radiolabeled, showing that the retarded band is not due to any structural artifact of either oligomer. The RY28 alone is not shifted even in the presence of the peptide.

The structure was finally probed with KMnO_4_ and DEPC modifications followed by sequencing. Ideally, chemical modification is performed on an isolated triplex, which in our study proved difficult due to the small size and low stability of the complex. We therefore carried out modification reaction on the annealed samples in the presence of the peptide. We are aware that during annealing various species are under equilibrium, and presence of such alternative structures will interfere with the band pattern of the RY28 hairpin molecule in isolation or in the triplex. We ensured that the association reaction between RY12 and RY28 was complete (as indicated by our thermal denaturation data) before precipitation and chemical treatments. It is reasonable to expect that under optimal experimental conditions the intense bands would be from modification of the major species of our interest and we may be able to detect a footprint for the triplex. Figure 4A shows the results of KMnO_4_ modification of several samples that were subjected to same treatment throughout. Unlike in EMSA we observed a complete protection of chemical groups in case of footprinting experiment suggesting the formation of stable triplex in presence of REWER. This discrepancy may be due to the fact that EMSA is a non-equilibrium method, whereas in footprint assay components of triplex are in binding equilibrium when treated with KMnO_4_ and DEPC [27]. Figure 4A, lane 1 shows result for RY28 hairpin alone. Due to their positions in loop of the RY28 hairpin T14, T15 and possibly T12 present at loop closure are expected to be modified by KMnO_4_. We observed that these bands are intense. Additionally prominent bands due to T4, T6 and T10 were also observed. The later three thymines on the 5′-arm of the hairpin are hypersensitive but the corresponding thymines on the 3′ -arm are not. This asymmetric pattern can be explained if there are kinks at the junctions of purine and pyrimidine stretches exposing the thymines on one arm and burying the corresponding thymines on the opposite arm making them less sensitive [13]. Almost identical band pattern resulted upon KMnO_4_ modification of a 1∶1 mixture of RY28 and RY12 (Figure 4A, lane 2) suggesting that they do not interact. KMnO_4_ sensitivity pattern of the hairpin molecule changes drastically in the presence of the pentapeptide, REWER (lane 3). The thymines on the 3′ -arm of the hairpin are now hypersensitive while those on 5′ -arm are not. We are unable to explain this observation except that it may be due to the binding of REWER to the hairpin. Following an earlier study with another peptide (KGWGK), REWER is expected to bind to RY12 duplex in the major groove resulting in the unwinding of the double helix by intercalating tryptophan [26]. When the triplex forms, i.e., with a 1∶1 mixture of RY28 and RY12 in the presence of REWER, thymines on both the arms of RY28 becomes insensitive to KMnO_4_ and a footprint develops. Figure 4A (lane 4) shows that only T15 in the loop, T6 and T4 are sensitive to KMnO_4_.

**Figure 5:**
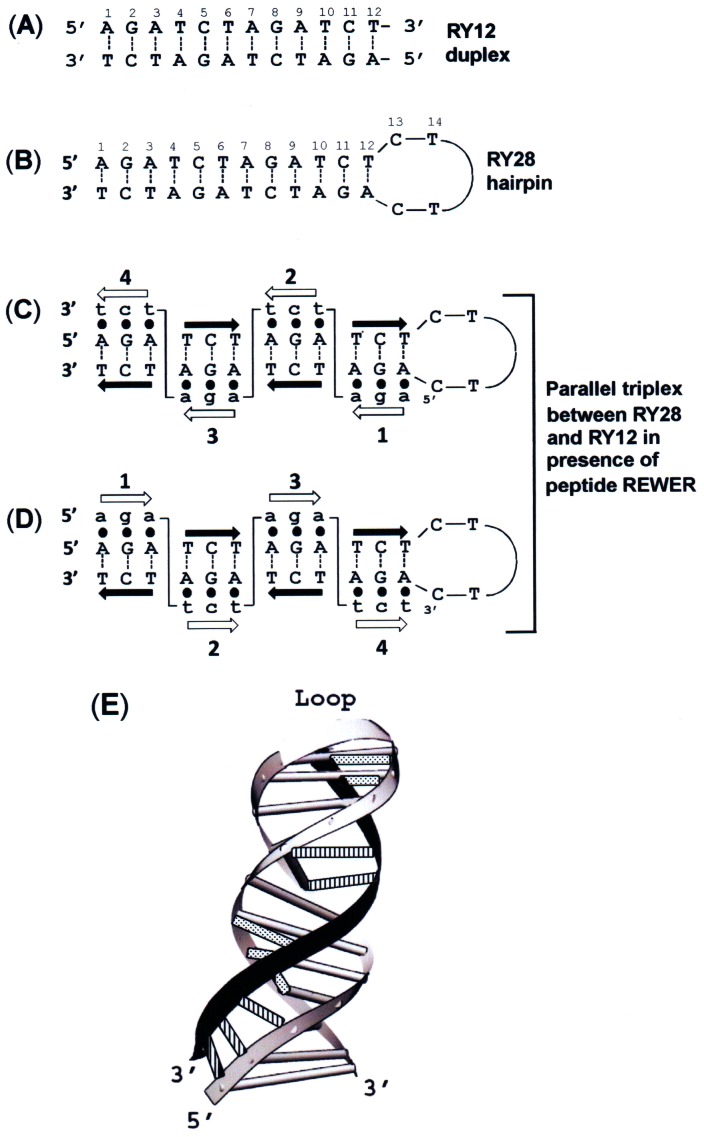
Schematic representation of the proposed triplex structure. **A**) Base sequence of RY12 dodecamer. **B**) RY28 hairpin. **C & D**) Schematics of base pairing in the triplex formed between RY12 and RY28. It show the representation diagram of the two putative triplex formations via alternate strand recognition (–) represent Watson-Crick base pair and (•) represent non Watson- Crick hydrogen bonds. The third strand is shown in lowercase. Open and solid arrows depict the polarity of third strand and hairpin duplex, respectively. Numbers 1–4 represent base triads in triplex structure. A parallel triplex is formed when three purines of the third strand form hydrogen bonds with underlying purine of the hairpin (Pu•PuPy) it then changes strand and binds to the other strand of the hairpin (Py•PuPy) in an antiparallel orientation. Please note that unlike more commonly found orientation in Pu•PuPy triplex where chemically homologous strands show antiparallel polarity our model suggests homologous strands in parallel orientation. **E**) A three dimensional rendition for a triplex of type **C**) in which the third strand recognizes alternate strands of a hairpin duplex. Shaded bars in the hairpin structure represent Watson-Crick hydrogen bonding. The third strand is shown in the middle as black ribbon. The purine triplet of the third strand (e.g., aga) forms base pairs (dotted bars) with the purine tract (e.g., AGA) of one strand of the Watson-Crick hairpin, whereas the pyrimidine triplet of the third strand (e.g., tct) base pairs (vertical bars) with the purine tract (e.g., AGA) of the other Watson-Crick strand.

Consistent with the results of KMnO_4_ modification, DEPC modification occurred on adenines and guanine of the RY28 hairpin (Figure 4B, lane 2). This pattern is not changed when DEPC modification is done on an annealed 1∶1 mixture of RY12 and RY28 (Figure 4B, lane 3). It should be noted that the adenines, incidentally, are at the junctions of the stretches of purines and pyrimidines. DEPC is known to carboxylate bases at the B-Z junction [24], and at cruciform loops [25]. In the triplex, however, all the adenines are insensitive to DEPC and a triplex footprint is observed (Figure 4B, lane 4). This result although similar to that of KMnO_4_ reaction, seemed surprising since the N7 of purines in a parallel triplex are expected to be free and sensitive to DEPC. In a previous study authors showed that with deproteinised joint molecules from RecA recombination, the N7 of adenines and guanines on the anticomplimentary strand were sensitive to DEPC and those on other strands remained insensitive [28]. In our proposed triplex structure, because of the palindromic nature of the sequence there is no specific anticomplementary strand and the observed bands due to A7, A9 and A17 are possibly due to contamination of minor species of malformed triplex as judged by the intensity of these bands when compared to the other lanes. It should be noted, however, that Chiu et al., used the deproteinised molecule [28], which was possibly a collapsed triplex, whereas in the present study triplex is bound to the peptide, which may have blocked the N7 in the major groove. Thus the results of chemical probing indicate that the bases in our triplex are inaccessible to KMnO_4_ or DEPC, which is consistent with the triplex structure proposed here. These results prompted us to conclude that RY28 hairpin joins with a single strand of homologous RY12 to form triple helix. Figure 5C & D shows the schematic diagram of the two possible putative triplex formations via alternate strand recognition. In this complex, triplex involving Pu•PuPy type (Figure 5C&D triads 1&3) both purine strands have the same polarity and are parallel whereas triplex involving Py•PuPy type have third strand in antiparallel orientation (Figure 5C&D triads 2&4). A shifted antiparallel triplex with a three base shift can also be conceived, but thermal stability data and chemical probing results as discussed below do not support such a structure.

The present investigation on possibility of triplex formation between RY28 hairpin and a single strand of RY12 in the presence of REWER, was prompted by an unusual observation of a triphasic melting curve of RY12 and RY28 in presence of REWER, which was not observed in the presence of KGWGK [26]. We believe this difference in the behavior of the two peptides is due to their sequence difference as well as the differences in the extent of unwinding they produce by tryptophan intercalation. Amino acids side chain intercalation is an important parameter for homologous pairing. However, if the pentapeptide binds in the major or minor groove of DNA, invasion by the third strand to form the triplex must occur in the other unoccupied groove. Both the grooves thus being blocked, the bases of RY28 hairpin would be protected from KMnO_4_ or DEPC modification resulting in footprint of the triplex as observed here. Present study do not conclusively prove the major or minor groove occupation of the third strand, it is interesting to note that our proposed triplex structure with tryptophan intercalation every three bps [26] is strikingly similar to the theoretically proposed model of minor groove associated triplex [29]. Although peptide is essential for triplex structure we do not know the exact mechanism of REWER-induced triple-helix formation. We do not know if the binding of REWER and oligonucleotides is of covalent or noncovalent type. Structural studies on our well-defined system by NMR or X-ray diffraction only can resolve this issue. Our system serves not only as a model for studying ligand-mediated triplex formation but also has a potential to be used as tool in molecular biology and biochemistry [5], [30], [31].
